# Effects of Promotional Materials on Attitudes and Fear towards Colorectal Cancer Screening among Chinese Older Adults: An Experimental Study

**DOI:** 10.3390/ijerph14070769

**Published:** 2017-07-13

**Authors:** Doris Y. P. Leung, Joanne M. T. Chen, Vivian W. Q. Lou, Eliza M. L. Wong, Aileen W. K. Chan, Winnie K. W. So, Carmen W. H. Chan

**Affiliations:** 1 The Nethersole School of Nursing, The Chinese University of Hong Kong, Hong Kong, China; jchen@cihe.edu.hk (J.M.T.C.); elizawong07@gmail.com (E.M.L.W.); aileenchan@cuhk.edu.hk (A.W.K.C.); winnieso@cuhk.edu.hk (W.K.W.S.); whchan@cuhk.edu.hk (C.W.H.C.); 2 School of Health Sciences, Caritas Institute of Higher Education, Hong Kong, China; 3 Department of Social Work and Social Administration, The University of Hong Kong, Hong Kong, China; wlou@hkucc.hku.hk

**Keywords:** colorectal cancer, screening, screening interest, cancer fear

## Abstract

Colorectal cancer (CRC) screening is a cost-effective prevention and control strategy. However, the promotion of CRC screening for older adults may be difficult because reading CRC prevention information may evoke embarrassment, fear, and anxiety towards the screening procedure and cancer diagnosis. This study aims to (1) examine the effects of three promotional materials for CRC screening on the attitudes toward CRC screening tests (screening interest, screening effectiveness, and trust in the screening results) and cancer fear, and (2) to explore the interaction effect of cancer fear with screening effectiveness and trust in the screening results on screening interest of the three screening tests (fecal occult blood test (FOBT), flexible sigmoidoscopy, and colonoscopy) among Chinese older adults. A total of 114 community-dwelling older adults were asked to look at the corresponding promotional materials (pamphlet, cartoon, and video) of one of the three study groups. The pamphlet and video represent convention strategies and the cartoon represents an innovative strategy. No significant difference was observed in the screening interest and cancer fear across groups. FOBT was the most preferred screening modality. The video group has a large proportion agreed screening effectiveness of flexible sigmoidoscopy than pamphlet and cartoon groups and trusted in the screening results for FOBT and flexible sigmoidoscopy than the pamphlet group. Logistic regression results showed that the effect of trust in the screening results on screening interest for colonoscopy was greater among participants with higher cancer fear than those with lower cancer fear level. In conclusion, the three promotional groups had produced similar results in their attitudes toward CRC screening and cancer fear. The use of cartoons may be a comparable approach with conventional methods in the promotion of CRC screening. Additional components that can arouse fear and boost response efficacy simultaneously might also be useful for the effective promotion of colonoscopy among Chinese older adults.

## 1. Introduction

Colorectal cancer (CRC) is a worldwide public health challenge, which has the fourth highest cancer-related mortality rate with an estimated 694,000 people dying from CRC in 2012 [1]. CRC is highly treatable in its early stage. Thus, CRC screening presents a cost-effective prevention and control strategy because early detection and treatment of CRC among asymptomatic patients can result in a significant reduction in mortality rate [2,3]. Statistical simulation models using data collected from 1975 to 2006 in US have also shown that the decrease in CRC rates can be majorly attributed to screening rather than the decrease in risk factors [4]. Despite the dramatic improvements in cancer prevention and control, CRC-related medical problems are still challenging—especially in the Asian region, including Hong Kong—because the incidence and mortality rates of CRC have increased tremendously in the last two decades [5]. Yet, previous studies have showed that Chinese people tended not to take any action even they are provided with the service free of charge or at a reduced cost [6,7].

Reading CRC prevention information may evoke embarrassment, fear, and anxiety toward the screening procedure and cancer diagnosis because of the threatening nature of cancer and unpleasant screening procedures. In our culture, Chinese people, particularly older adults, may find CRC screening tools uncomfortable because the procedures are unpleasant. For example, they may refuse to talk about bowel because they consider it as “dirty”. These negative attitudes may hinder people, particularly older adults, from taking the next important step, which is cancer screening [8]. According to Witte’s Extended Parallel Processing Model (EPPM) [9], when fear is aroused after reading a promotional material, two possible reactions are expected from the individuals. First, individuals are more likely to engage to danger-control process when they perceive that the method is effective or they have the self-efficacy to control health threat by undergoing the method. Second, individuals are more likely to engage in fear-control process when they perceive that the method is ineffective or they do not have sufficient self-efficacy by denying and avoiding the process.

Numerous systematic reviews of demographic, social, and environmental determinants of CRC screening have been produced in the last few decades [10,11,12,13,14,15]. The single systematic review of 83 studies focusing on people aged ≥65 revealed a number of demographic variables (educational level, race, ethnicity, gender, marital status, and living status) and health-care systems related variables (recommendation by physicians, having a usual source of care, and lack of health insurance) were important factors to the uptake of CRC screening tests [13]. However, only a few of the studies in this review examined screening test related factors, such as perceived screening effectiveness. Another recent review on Chinese adults also reported similar factors that influence CRC screening behavior [15]. As noted in this recent review, only a few of the reviewed studies utilizing current health theories to guide the inquiry.

Moreover, the findings from previous research on risk communications are also mixed: the provision of knowledge may not be as effective in motivating people to participate in cancer screening as expected. A previous study in the US reported that participants felt knowledgeable and relieved after reading printed CRC prevention information [16]. Contrarily, a population-based study showed that providing information about the risks of cancer screening indeed decreased the participants’ assessment of the decision-making process in taking screening tests [17]. Another experimental study found that people process risk information affectively and cognitively. Mostly, these emotional reactions can be even more influential than factual knowledge in making decisions regarding preventive behaviors [18]. Thus, the feelings of the recipients toward risk communication process, which is an important element in decision making, must be understood. However, most of these materials were prepared in technical medical terms, which are not specifically designed for people with limited health literacy. Older people usually have a lower level of health literacy and may find the content of these materials difficult to understand. As a result, the messages presented in the materials may not be well received. Thus, searching for innovative strategies, such as cartoons without technical medical terms, is important to communicate the importance of CRC screening that can foster decision making regarding this preventive behavior.

In Hong Kong, a pilot program for CRC screening with fecal immunochemical test provided at a reduced cost for adults between the ages of 61 and 70 was launched in September 2016 [19]. Before the launch of this pilot program, CRC screening is opportunistic and people who want to undergo the process must pay. The estimated CRC screening rate among older adults ranged from 12% to 25% [20,21]. The three screening methods given in the promotional material included FOBT, flexible sigmoidoscopy, and colonoscopy [22]. No local clinical guideline for CRC screening was published by medical professional organizations for the general population although an expert working group under the Government of the Hong Kong Special Administrative Region (SAR) in 2012 suggested that people at the age of 50–75 should seek information regarding CRC screening from physicians [23].

Understanding the attitudes of people after their exposure to cancer prevention information is a fundamental and crucial step to improving the effectiveness of promotional interventions on screening behavior. This study thus had two aims: (1) to compare attitudes (screening interest, screening effectiveness, and trust in the screening results) towards CRC screening methods and cancer fear after looking at the three existing CRC promotional materials (pamphlet, cartoon, and video), and (2) to explore the interaction effects of cancer fear with screening effectiveness and trust in the screening results on screening interest for the three CRC screening methods, (FOBT, flexible sigmoidoscopy, and colonoscopy) among Chinese older adults.

## 2. Materials and Methods

### 2.1. Study Design and Participants

This study is a three-armed quasi-experiment. Three study groups (pamphlet, cartoon, and video) were created. According to Hertzog’s recommendation for pilot studies [24], 30–40 participants per arm are needed to obtain a direct estimate of a between-group effect size for a subsequent power analysis. We planned to recruit eight participants in each of the four gender and education cells, resulting in 32 participants in each of the study groups to have a balance sample in gender and educational level (≤primary education vs. >primary education).

A convenience sample of older people was recruited from three community centers for older people in Hong Kong from January to April 2014. Participants were eligible if (1) they aged 60 or above and (2) intact cognitive function as measured by Mini-Mental State Examination (MMSE) score ≥18 [25]. Participants were excluded if they (1) were currently diagnosed with a mental disease (e.g., schizophrenia or schizoaffective disorder), had a severe cognitive impairment (e.g., dementia or Alzheimer’s disease), or a history of stroke; (2) could not communicate in Cantonese or Mandarin; or (3) had hearing or visual impairment. The upper age limit for the participants were not restricted because no CRC clinical guideline and screening program existed in Hong Kong when the study was conducted. The study was approved by the Institutional Review Board of Joint-Chinese University of Hong Kong-New Territories East Cluster of Hong Kong Hospital Authority (CRE-2013.558), and informed consent was obtained from all subjects before their participation.

### 2.2. Procedure

The sample included older adults who visited the community centers for older people of three non-governmental organizations (NGOs), in which older adults usually have their daytime social activities. Four research assistants (RAs), in which one (RA1) is responsible for subject recruitment and each of the remaining three (RA-Is) delivers one of the three interventions separately, were trained for the study. After a scheduled session of regular social activities of the NOG, the older adults were approached by RA1 for eligibility screening. After obtaining written consent, eligible participants completed a self-administered questionnaire on their demographic information and prior knowledge of CRC. Based on gender and educational level of the participants and the availability of RA-Is, the RA1 assigned the participants to the respective study group to read the printed material or watch the video in a laptop computer for the information regarding CRC and its screening procedure for the respective study group provided by the RA-I in the NGO. Following looking at the promotional material, the participants immediately completed a questionnaire measuring their attitudes towards screening and collecting comments regarding the promotional materials. Participants could ask the RA for assistance in filling the questionnaires if needed. Participants who have completed the survey were given an incentive of HK$20 in value for their time to boost participation.

### 2.3. Promotional Materials of CRC Screening

In this study, three CRC promotion information materials were used, including (1) the 11-page pamphlet from the Department of Health, HKSAR (pamphlet group) [22]; (2) a 30-s video in cartoons developed by the Colorectal Cancer Awareness Campaign funded by the Cancer Foundation Hong Kong (cartoon group) [26]; and (3) a 3-min self-developed nurse-delivered promotional video (video group) (Table 1). The 11-page pamphlet covers the following topics: (1) CRC prognosis, (2) CRC statistics, (3) CRC symptoms, (4) CRC risk factors, (5) CRC screening tests, (6) benefits and potential harms of CRC screening, and (7) CRC treatments. The nurse-led video is an audio version of the 11-page pamphlet and a demonstration of a stool sample preparation for FOBT using clay. The cartoon video by the CRC Awareness Campaign uses cartoons to illustrate the prognosis of CRC, implying that polyps should be removed when they have developed to an advanced stage. The promotional materials, in the forms of pamphlet and nurse-led video, represent the conventional strategy by providing disease-related knowledge, while the cartoon video is an innovative strategy of health promotion.

### 2.4. Measures

Following a previous study [27], attitudes towards CRC screening (screening interest, screening effectiveness, and trust in the result of screening) was measured by three close-ended questions for each of the three CRC screening tests as stated in the pamphlet [22]: FOBT, flexible sigmoidoscopy, and colonoscopy. The three close-ended questions are:

(1) Screening interest: “If you were offered a CRC screening test, would you take up the offer?” measured by a four-point Likert scale ranging from ‘Definitely Not’ to ‘Definitely Yes’;
(2) Screening effectiveness: “The bowel screening is effective in detecting bowel cancer” measured by a five-point Likert scale ranging from ‘Strongly Disagree’ to ‘Strongly Agree’; and
(3) Trust in the results of screening: “If you completed a screening test, would you trust the results of the test?” measured by a four-point Likert scale ranging from ‘Not At All’ to ‘Very Much’.

For each of the three types of CRC screening tests, three dichotomous variables were created:

(i). Screening interest was coded as “Yes = 1” when the participant answered “Definitely Yes” or “No = 0” for the other three options,
(ii). Screening effectiveness was coded as “Yes = 1” when the participant answered “Strongly Agree” or “No = 0” for other options,
(iii). Trust in the screening results was coded as “Yes = 1” when the participant answered “Very Much” or “No = 0” for other options.

Cancer fear, defined as emotional response to CRC and its screening behaviors, was measured using the eight-item Chinese Colorectal Cancer Fear Scale, which has been validated in the Chinese population [28]. The cancer fear score ranges from 1 to 5. A higher score indicates that the participant is more fearful about CRC. Cronbach’s alpha value was 0.94 for cancer fear in the present study.

Demographic information and covariates: we collected demographic variables, including age, gender, education level, marital status, financial status, cognitive functioning (MMSE), status of CRC screening, whether someone you knew has undergone a CRC screening test, and presence of chronic diseases. A list of 28 chronic diseases common in older people, such as hypertension, diabetes, lung disease, and heart disease, was presented for selection. Prior knowledge of CRC was measured using a validated questionnaire that measures knowledge about the symptoms and risk factors of CRC from a previous study in Hong Kong [29]. Participants were asked two questions: “What are the symptoms of bowel cancer?” and “What are the risk factors of bowel cancer?” The list of answers was not disclosed to the participants, and the numbers of correct answers for these questions were counted.

### 2.5. Statistical Analysis

Descriptive statistics, including frequencies and percentages for categorical data and means and standard deviations for continuous data, were calculated to describe the sample characteristics. ANOVA for continuous variables and chi-square tests for categorical variables compared baseline characteristics across study groups. Chi-square tests compared the effect of three promotional materials on attitudes towards the three CRC screening methods, and ANOVA compared the effect for cancer fear. Pairwise comparisons were then performed if there was a significant difference in the outcome variable. For each of the three screening methods, logistic regression models with two blocks were used to examine the dual process suggested by the EPPM [9] to identify the associated factors of screening interest. The variables in Block 1, including demographic characteristics and intervention type, were treated as independent variables, and backward elimination was applied to retain potential associated factors (*p* < 0.05). Then, as guided by the EPPM [9], the variables in Block 2, including screening effectiveness, trust in the screening results, and cancer fear, and the interaction terms of cancer fear with screening effectiveness and trust in the screening results, were included in the final logistic regression model. Odds ratio and its 95% confidence intervals were reported in the logistic regression results. The significance level of the statistical tests was set at 5% whenever applicable. All the analyses were conducted using SPSS version 22.0 (IBM Corporation; Armonk, NY, USA).

## 3. Results

A total of 114 older people were recruited in the study (pamphlet: 39, cartoon: 39, and video: 36). Among them, the mean age was 74.2 ± 11.5 years, 42.1% were male and 58.8% had no formal education or had below primary education. Their mean MMSE score was 25.7 ± 3.3, and the mean number of chronic disease was 1.8 ± 1.5. They had low mean scores in terms of knowledge about CRC symptoms and risk factors. 25.4% had CRC screening before, and 21.9% knew someone who had CRC screening. No statistical significant differences were observed in the demographic characteristics among the three groups (Table 2).

The results reported in Table 3 revealed that significant differences were observed in the proportions of participants, agreeing with the screening effectiveness for flexible sigmoidoscopy (pamphlet: 43.6%, cartoon: 59.0%, video: 80.6%, *p* = 0.004), and trust in the results of FOBT (pamphlet: 7.7%, cartoon: 23.1%, video: 30.6%, *p* = 0.04), and of flexible sigmoidoscopy (pamphlet: 25.6%, cartoon: 46.2%, video: 52.8%, *p* = 0.04). Further analyses revealed that a significantly higher proportion in screening effectiveness for flexibility sigmoidoscopy was observed in the video group when compared with the pamphlet group (*p* = 0.002) and the cartoon group (*p* = 0.049). The significant differences in trust in the screening results of FOBT (*p* = 0.017) and of flexible sigmoidoscopy (*p* = 0.019) was between the video and the pamphlet groups. No statistically significant differences in screening interest and cancer fear among the three groups were found.

Table 4 shows the results of the logistic regression of screening interest for each of the three CRC screening methods. For FOBT, all of the variables in Block 2 was insignificant, and two demographic characteristics of the participants, namely, “knowledge about CRC symptoms” (OR = 1.28; 95% CI: 1.00–1.64) and “knew someone had CRC screening” (OR = 7.2; 95% CI: 1.84–28.0), were positively associated with the likelihood of having screening interest. For flexible sigmoidoscopy, only screening effectiveness (OR = 4.7; 95% CI: 0.84–26.8) in Block 2 and “had CRC screening before” in Block 1 (OR = 3.9; 95% CI: 1.4–10.8) were positively and significantly associated with screening interest. For colonoscopy, screening interest was negatively associated with cancer fear (OR = 0.86; 95% CI: 0.76–0.99). The interaction of cancer fear and trusts in the screening results was also statistically significant with a coefficient of 1.19, indicating that the trust in the screening results affects the likelihood of having greater screening interest among participants with higher cancer fear than those with lower cancer fear. In addition, “had CRC screening before” (OR = 3.7, 95% CI: 1.19–11.6) and “being in the cartoon group” (OR = 4.0; 95% CI: 1.16–14.1) were positively associated with screening interest.

## 4. Discussion

The results in the current study showed that the three promotional materials on CRC screening had similar effects in the attitudes towards CRC screening and cancer fear regardless of the screening method used. However, significantly greater proportions of participants in the video group showed screening effectiveness to flexible sigmoidoscopy and colonoscopy, and trust in the screening results in FOBT and flexible sigmoidoscopy. Although no differential effect is observed on screening interest, regardless of the intervention type, greater proportions (>44%) of our participants showed interest in undergoing FOBT among the three screening tests. This finding was in line with previous studies, showing that FOBT is a more preferred screening modality among Asian-Americans [30,31,32]. FOBT is a more affordable test and is non-invasive in nature, suggesting that FOBT may be the best initial test that should be promoted to older Chinese adults. Another interesting finding was the great discrepancy between the proportions in screening effectiveness and screening interest for flexible sigmoidoscopy and colonoscopy, but not for FOBT in the video group when compared with the pamphlet and cartoon groups. A possible explanation for the high proportions in screening effectiveness for flexible sigmoidoscopy and colonoscopy in the video group is that effectiveness for these two screening methods were emphasized in the corresponding video; hence, the participants might have memorized and reported the fact correctly in the post-intervention questionnaire. Although a nurse, as a health provider, delivered the intervention in the video group, the proportions of screening interest for flexible sigmoidoscopy and colonoscopy were very low when compared with that in FOBT, whereas a reverse pattern was observed for screening effectiveness. It is possible that the participants in the video group are not convinced of their self-efficacy in undergoing these two screening actions because no demonstrations were shown in the nurse-delivered video; hence, they have low rates in screening interest [33] or they have perceived no threat and do not need any follow-up action [9]. However, one key message from this result is that the effect of a healthcare provider in promoting behavioral changes without direct contact with older adults is doubtful.

Although comparable to previous studies for Asian-Americans and Chinese-Americans (53.2–60%) [30,31,32,33,34], the post-intervention proportions of participants had interest in CRC screening in the current study were still considered low (19–59%). A possible explanation for the low screening interest rates is the lack of provider–patient communication in the three interventions. A meta-analysis of eight studies reported that communication with a medical provider was a strong associated factor of the likelihood of CRC screening among Chinese adults (pooled OR = 7.99) [15]. Another review on 35 studies that examine cancer adherence concluded that provider recommendation is a necessary component, although not sufficient, for adherence to cancer screening guideline [35]. Another possible explanation for the low screening interest in our participants may be the opportunistic nature for CRC screening in Hong Kong, causing participants to have low interest in screening because of financial concern. Nevertheless, the current findings highlight that the messages from the promotional materials might not be well received by the participants, and adopting more proactive health promotional strategies, such as the use of lay health workers as health providers, may be needed to recommend CRC screening tests to enhance direct contact with participants [34,36,37].

The three promotional groups had produced similar results in attitudes towards CRC screening and cancer fear. Although the materials in the cartoon group only cover the prognosis of CRC and the necessity of polyp removal, the coverage is lower when compared with the pamphlet and video groups. It is possible that just by showing the growing stage and the visual removal of polyps in a soft tone might have already provoked CRC awareness among the participants. Alternatively, considering that more than half of our participants had educational level of primary school or below, their level of health literacy might be insufficient to understand the technical terms and the abstract ideas presented in the pamphlet and the nurse-led video. Nevertheless, the use of cartoons without technical medical terms may be a potential effective approach to demonstrate the basic concepts of polyp and its growth, and convey the message of the importance of having these procedures to increase screening interest among Chinese older people when the screening for CRC is opportunistic. However, the result is subject to a certain amount of errors, and thus, is only preliminary.

The negative effect of cancer fear in screening interest for colonoscopy after reading promotional materials were consistent with the results in the previous studies [17,18]. The finding on the significant interaction term of cancer fear and trust in screening results provides some support to the prediction of the danger-control process in EPPM for CRC screening [38]. These findings provide preliminary support to the claim of the non-linear association of fear with screening behavior [39]. However, this phenomenon was observed only for colonoscopy and not for FOBT and flexible sigmoidoscopy. A previous study showed that many Chinese-Americans considered colonoscopy a diagnostic procedure to investigate and treat pre-malignant lesions [40]. Furthermore, compared with FOBT and flexible sigmoidoscopy, colonoscopy is a more invasive procedure that might have aroused fear in some of the participants. Nevertheless, in line with Liu et al.’s study [16], our participants in all the three groups had low mean cancer fear levels for all the three screening tests. These results were comparable with that of the participants in a local study who did not receive intervention [41], suggesting that the fear level of most of our participants might not be high enough to motivate them to think about the necessity of taking further action [38]. Again, the finding is also subject to a large extent of errors.

The current study had a number of important limitations. In this experimental study, the sample size was small; thus, the power of the statistical tests was affected. The results should be interpreted with caution, considering the potential large Type II error of the statistical tests, and the study should be replicated with larger sample sizes. In addition, the pre-test scores of attitudes towards CRC screening and cancer fear were not measured before the experiment, which might have induced bias in the findings because it precludes us from measuring the changes in these outcome variables after the interventions. The use of the cut-off point of 18 in MMSE scores for cognitive functioning might have included some older adults with mild to moderate undiagnosed dementia. However, according to the cut-off points of MMSE in the Chinese population [25], only six participants with primary education had MMSE scores lower than 21, and three participants with some secondary education had MMSE scores lower than 25, resulting in a small proportion of 7.9% of potential undiagnosed dementia. The upper limit in age for the eligibility of participants was not set, which is another limitation. Very old people may not be interested in taking any preventive measure for CRC because of their short life expectancy. In addition, CRC screening was opportunistic when the study was conducted. Thus, the current findings might not be applicable to the situation of organized screening programs, in which screening is usually available free of charge or at a reduced cost. Furthermore, some of our participants indicated that they had CRC screening before. Although no significant difference was observed among groups, the current results will definitely be affected. The effects of CRC screening were controlled when the interaction effect of cancer fear with screening efficacy-related variables on screening interest in the logistic regression were examined. Moreover, the use of convenience sampling will limit the generalizability of the findings. Our participants were not assigned randomly, which might have induced bias in the study findings, although no significant group difference in the demographic characteristics of the participants at the baseline was observed. Furthermore, the measures of self-efficacy were not included in performing the desirable action, which preclude us from exploring the potential mechanisms on the motivation to participate in CRC screening, further using promotional materials.

## 5. Conclusions

The findings from this study show that although no differential effect was observed among the three interventions in the promotion of CRC screening interest among Chinese older adults, FOBT was the most preferred screening modality. The use of a cartoon may be an effective approach to promote screening interest in colonoscopy, and recommendation from healthcare professionals without direct contact might not be effective in promoting CRC screening. Healthcare professionals may consider delivering educational materials on CRC screening to older people with direct contact supplemented with cartoon to illustrate CRC prognosis. In addition, the study findings highlight the role of cancer fear and trust in the screening result in screening interest, particularly for colonoscopy. Additional components that can arouse fear and boost response efficacy simultaneously to the interventional materials that are targeted and tailored for this particular subgroup could be beneficial for an effective promotion of CRC screening.

## Figures and Tables

**Table 1:** Content of the three CRC promotional materials.

Topic	Content	Pamphlet	Cartoon	Video
1. Prognosis of CRC	The development of CRC	Yes	Yes	Yes
2. Statistics of CRC	Incidednce; and age-secific and gender-specific mortality in Hong Kong			
3. Symptoms of CRC	Common symptoms of CRC such as blood in stool, change in bowel habits and weight loss	Yes	No	Yes
4. Risk factors of CRC	Common risk factors such as lifestyles, age, and family history	Yes	No	Yes
5. CRC screening tests	Fecal occult blood test, flexible sigmoidoscopy, and colonoscopy	Yes	No	Yes
6. Demonstration of FOBT	The process of preparation of the stool sample using clay	No	No	Yes
7. Benefits and potential harms of CRC screening	Early detection and treatment of CRC; False alarm of CRC	Yes	No	Yes
8. Treatments of CRC	Possible medical treatments of CRC and maintaining a good lifesty; Contact information of organizations providing support to CRC survivors	Yes	No	Yes

**Table 2:** Characteristics of study participants.

Variable	Total (*n* = 114)	Pamphlet (*n* = 39)	Cartoon (*n* = 39)	Video (*n* = 36)	*p*
Age, Mean ± SD	74.2 ± 11.5	71.6 ± 14.2	76.3 ± 8.1	74.6 ± 10.9	0.2
MMSE, Mean ± SD	25.7 ± 3.3	26.5 ± 3.5	25.3 ± 3.0	25.7 ± 3.3	0.2
Male, *n* (%)	48 (42.1)	15 (38.5)	16 (41.0)	17 (47.2)	0.7
No formal education/below primary school level, *n* (%)	67 (58.8)	22 (56.4)	24 (61.5)	21 (58.3)	0.9
Married/cohabitation, *n* (%)	73 (64.0)	23 (59.0)	29 (74.4)	21 (58.3)	0.2
Family income ^1^, Mean ± SD	2.9 ±2.6	3.3 ± 2.8	2.8 ± 2.7	2.6 ± 2.2	0.4
Mean number of chronic disease, Mean ± SD	1.8 ± 1.5	1.5 ± 1.2	2.0 ± 1.7	1.8 ± 1.6	0.4
Knowledge about CRC symptom, Mean ± SD	1.8 ± 2.0	2.00 ± 2.0	1.7 ± 1.9	1.7 ± 2.2	0.9
Knowledge about CRC risk factors, Mean ± SD	2.2 ± 2.6	2.6 ± 2.7	2.0 ± 2.4	1.8 ± 2.6	0.4
Had CRC screening before, *n* (%)	29 (25.4)	13 (33.3)	7 (17.9)	9 (25.0)	0.3
Knew someone had CRC screening, *n* (%)	25 (21.9)	7 (17.9)	11 (28.2)	7 (19.4)	0.5

^1^ Response for Family Income: 1 = <HKD5000; 2 = HKD5000–HKD9999; 3 = HKD10,000–HKD14,999, 4 = HKD15,000–HKD19,999, 5 = HKD20,000–HKD24,999, 6 = HKD25,000–HKD29,999, 7 = HKD30,000–HKD39,999, 8 = ≥40,000.

**Table 3:** Results on attitudes towards CRC screening methods and cancer fear by group.

Variable	Pamphlet (*n* = 39)	Cartoon (*n* = 39)	Video (*n* = 36)	*p*			
				Overall ^1^	Pairwise Comparison ^2^		
					Pamphlet vs. Cartoon	Pamphlet vs. Video	Cartoon vs. Video
**Screening interest**							
FOBT, *n* (%)	23 (59.0)	18 (46.2)	16 (44.4)	0.38			
Flexible Sigmoidoscopy, *n* (%)	9 (23.1)	15 (38.5)	7 (19.4)	0.15			
Colonoscopy, *n* (%)	10 (25.6)	15 (38.5)	7 (19.4)	0.17			
**Screening effectiveness**							
FOBT, *n* (%)	13 (33.3)	17 (43.6)	21 (58.3)	0.09			
Flexible Sigmoidoscopy, *n* (%)	17 (43.6)	23 (59.0)	29 (80.6)	0.004	0.3	0.002	0.049
Colonoscopy, *n* (%)	23 (59.0)	25 (64.1)	29 (80.6)	0.12			
**Trust in the results of screening**							
FOBT, *n* (%)	3 (7.7)	9 (23.1)	11 (30.6)	0.04	0.1	0.02	0.5
Flexible Sigmoidoscopy, *n* (%)	10 (25.6)	18 (46.2)	19 (52.8)	0.04	0.1	0.02	0.6
Colonoscopy, *n* (%)	17 (43.6)	20 (51.3)	18 (50.0)	0.77			
Cancer fear, Mean ± SD	3.30 ± 0.96	3.17 ± 1.08	2.96 ± 1.00	0.34			

^1^ Chi-square test for proportion and ANOVA for continuous variable. ^2^ Pairwise comparisons of the outcome variable across study group.

**Table 4:** Logistic regression analysis for screening interest of FOBT, flexible sigmoidoscopy and colonoscopy.

Factors	FOBT			Flexible Sigmoidoscopy			Colonoscopy		
	OR	95% CI	*p*	OR	95% CI	*p*	OR	95% CI	*p*
**Block 1 variables**									
Number of chronic diseases	0.75	0.54–1.02	0.070						
Knowledge about CRC symptoms	1.28	1.00–1.64	0.049				1.28	0.99–1.67	0.06
Had CRC screening before				3.9	1.4-10.8	0.007	3.7	1.19–11.6	0.02
Knew someone had CRC screening	7.2	1.84–28.0	0.005						
Intervention									
Video							1		
Pamphlet							1.70	0.44–6.5	0.4
Cartoon							4.0	1.16–14.1	0.03
**Block 2 Variables**									
Cancer fear	0.95	0.89–1.02	0.2	0.95	0.83–1.10	0.5	0.86	0.76–0.99	0.03
Screening effectiveness	1.55	0.14–17.1	0.2	4.7	0.84–26.8	0.04	1.72	0.28–10.4	0.5
Trust in the screening results	0.15	0.004–5.0	0.3	0.10	0.01–7.1	0.3	0.05	0.01–2.96	0.2
Cancer fear × Screening effectiveness	0.91	0.77–1.07	0.3	0.90	0.79–1.04	0.1	0.98	0.85–1.12	0.7
Cancer fear × Trust in the screening results	1.08	0.95–1.23	0.2	1.13	0.95–1.34	0.2	1.19	1.01–1.41	0.04
Nagelkerke R^2^	0.325			0.284			0.328		
